# A target trial emulation of dexmedetomidine to treat agitation in the intensive care unit

**DOI:** 10.62675/2965-2774.20250010

**Published:** 2025-03-14

**Authors:** Ary Serpa, Marcus Young, Atthaphong Phongphithakchai, Akinori Maeda, Yukiko Hikasa, Nuttapol Pattamin, Nuanprae Kitisin, Gehan Premaratne, Gabriel Chan, Joseph Furler, Meg Stevens, Dinesh Pandey, Hossein Jahanabadi, Yahya Shehabi, Rinaldo Bellomo

**Affiliations:** 1 Department of Intensive Care Austin Hospital Melbourne Australia Department of Intensive Care, Austin Hospital - Melbourne, Australia.; 2 Department of Critical Care Melbourne Medical School University of Melbourne Melbourne Australia Department of Critical Care, Melbourne Medical School, University of Melbourne, Austin Hospital - Melbourne, Australia.; 3 Business Intelligence Unit Austin Hospital Melbourne Australia Business Intelligence Unit, Austin Hospital - Melbourne, Australia.; 4 Monash Health School of Clinical Sciences Monash University Melbourne Victoria Australia Monash Health School of Clinical Sciences, Monash University - Melbourne, Victoria, Australia.; 5 Australian and New Zealand Intensive Care Research Centre School of Public Health and Preventive Medicine Monash University Melbourne Australia Australian and New Zealand Intensive Care Research Centre, School of Public Health and Preventive Medicine, Monash University - Melbourne, Australia.

**Keywords:** Dexmedetomidine, Agitation, Delirium, Target trial emulation, Intensive care units

## Abstract

**Objective:**

Agitation is a major problem in the intensive care unit. However, no treatment has clearly emerged as effective and safe. Using target trial emulation, we aimed to test the hypothesis that early intervention with dexmedetomidine would accelerate agitation resolution.

**Methods:**

We read clinical notes in an electronic medical records system with natural language processing to identify patients with agitation. We obtained their demographics, trajectories, associations, and outcomes. We used g-formulas to study the possible effects of dexmedetomidine on agitation resolution and key outcomes.

**Results:**

We screened 7525 patients. Overall, 2242 patients (29.8%) developed within-intensive care unit agitation, and 2052 (27.3%) were eligible for inclusion in the target trial emulation, with 314 treated with dexmedetomidine. Dexmedetomidine-treated patients had more severe illness and were more likely to have unplanned emergency admissions with medical diagnoses. However, they achieved higher rates of resolution of within-intensive care unit agitation (94% *versus* 72%; *p* < 0.001) and lower 30-day mortality (5% *versus* 9%; p = 0.033). Early initiation of dexmedetomidine accelerated the resolution of agitation (risk ratio [RR] 1.13 [95%CI 1.03 - 1.21]; risk difference [RD] 9.8% [95%CI 2.6% - 15.4%]); extubation by Day 30 (RR 1.03 [95%CI 1.02 - 1.04]; RD 3.1% [95%CI 2.2% - 4.2%]); and reduced the chance of having a tracheostomy by Day 30 (RR 0.67 [95%CI 0.34 - 0.99]; RD -3.5% [95%CI -7.0% - -0.0%])

**Conclusion:**

Through target trial emulation analysis, early dexmedetomidine was associated with an increased rate of resolution of agitation and extubation and decreased tracheostomy risk.

## INTRODUCTION

Psychomotor agitation is a major and dangerous type of behavioral disturbance in intensive care patients.^1 , 2^ However, no treatment has been demonstrated to be effective in increasing its resolution and decreasing its associated adverse clinical outcomes in randomized trials.^3^

Haloperidol appears to be the most studied treatment for agitation.^4^ In support of its possible efficacy, in a randomized controlled trial (RCT) of prophylactic haloperidol, a lower proportion of patients developed agitation (assessed by the Richmond Agitation-Sedation Scale [RASS] score) than did those in the placebo group.^5^ On the other hand, once agitation occurs, no RCT has demonstrated that haloperidol treatment increases the speed or rate of its resolution or changes its associated outcomes.^4 , 6 , 7^

Given these limitations, alternative pharmacological options have been explored, with dexmedetomidine emerging as a potential agent for agitation management. Notably, none of the above studies included patients treated with dexmedetomidine. This is a major limitation because dexmedetomidine was shown to be superior to haloperidol in a single-center pilot comparative RCT of the treatment of agitated and ventilated intensive care unit (ICU) patients.^8^ Moreover, a multicenter double-blind RCT of dexmedetomidine for the treatment of agitated *delirium* in ventilated patients also revealed a faster time to extubation.^9^ Thus, dexmedetomidine may be a good option for controlling agitation. However, no RCT data exist on the possible efficacy of dexmedetomidine as a primary treatment for agitation in all ICU patients, including those not receiving mechanical ventilation (MV).

Ideally, the question of whether dexmedetomidine-based treatment of all ICU patients with agitation affects patient-centered outcomes should be studied in an adequately powered RCT. However, until such trials are available, clinical decision-making could be aided by well-conducted observational studies that explicitly mimic the strict design criteria of a clinical trial. Such studies can also be used to provide the basis for and inform the power and sample size of subsequent trials. Accordingly, we combined granular real-word data from a tertiary hospital in Australia with natural language processing (NLP) identification of agitation,^1 , 2^ and with the novel statistical technique of target trial emulation.^10 - 12^ We aimed to provide a framework for causal inference from observational data and to investigate the possible effects of early use of dexmedetomidine on the outcomes of ICU patients with agitation. We also aimed to test the primary hypothesis that dexmedetomidine would increase the odds of resolution of such agitation and the secondary hypothesis that it would also improve other relevant clinical outcomes.

## METHODS

### Study design

We conducted a target trial emulation comparing the effects of early dexmedetomidine on the outcomes of ICU patients with agitation.^10 - 12^ The details of the target trial emulation are presented in table 1 . Explicit emulation of a trial, particularly aligning the start of follow-up with the assignment of treatment strategies, eliminates the impact of immortal time bias, selection/survivor bias, and lead time bias.^10^ This study was approved by the Austin Hospital Human Research Ethics Committee (LNR/19/Austin/38), with a waiver of informed consent.

**Table 1:** Summary of the protocol of the target trial assessing the impact of dexmedetomidine in patients with agitation

Components	Hypothetical randomized trial	Emulation
Eligibility	All adult patients admitted to the ICUs of Austin Hospital, Melbourne between June 2016 and April 2021. Patients were eligible if they developed agitation during the ICU stay. Patients who started on dexmedetomidine before the first episode of agitation and patients with missing data for in hospital mortality were excluded	Same as the hypothetical trial
Treatment strategies	Treatment with dexmedetomidine Usual care without use of dexmedetomidine	Same as the hypothetical trial. In a sensitivity analysis, different moments of dexmedetomidine initiation after agitation onset were investigated
Treatment assignment	Patients are randomly assigned to one of the strategies at time zero. Stratification was performed based on clinical characteristics. The treatment team was aware of the assigned strategy	We assumed that patients were randomly assigned within levels of the baseline covariates
Follow-up	For each patient, follow-up starts at the time of assignment to a strategy (and all eligibility criteria are met) and ends at the resolution of agitation, occurrence of death or 30 days, whichever comes first	Follow-up starts at the first time when all eligibility criteria are met
Primary outcomes	30-day resolution of agitation	Same as the hypothetical trial
Causal contrast	Per protocol effect	Observational analog of the per protocol effect
Statistical analysis	In the per-protocol analysis, patients were censored when they deviated from their assigned strategy. The per-protocol effect was estimated after adjustment for baseline variables and for time-dependent variables associated with adherence to a strategy considering dexmedetomidine	Same as the hypothetical trial. The per-protocol effect was estimated under full adherence using the parametric g-formula*

ICU – intensive care unit. * More information available in the Supplementary Material .

### Patients

All adult patients (≥ 18 years old) admitted to the ICUs of Austin Hospital, Melbourne, between June 2016 and April 2021 were eligible for inclusion. If a patient had multiple admissions, only the first admission was considered. Patients were eligible if they developed agitation (see below for definition) during their ICU stay. Patients who started dexmedetomidine before the first episode of agitation and patients with missing data for in-hospital mortality were excluded. The baseline was defined as the first time that all eligibility criteria for the target trial emulation were met.

### Data collection and manipulation

All baseline and outcome data were collected from the Australian and New Zealand Intensive Care Society Adult ICU Patient Database run by the Centre for Outcome and Resource Evaluation.^13^ During the study period, according to ICU policy, patients received general care aimed at decreasing the risk of *delirium* , including frequent family visits, dimmed lights at night, minimal interaction to facilitate nighttime sleep cycling, and ensuring the use of spectacles and hearing aids as necessary.

Natural language processing techniques were used to extract the details of the assessments from the caregiver notes. Furthermore, the clinical progress notes of all caregivers were analyzed via NLP tokenizing techniques as previously described (natural language toolkit; NLTK 3.5).^14 , 15^ Each note was then searched for the presence of words, terms, or expressions, suggestive of agitation.^16^ Notes were completed at least every shift (8 hours), and additional notes could be written by different healthcare workers on the basis of patients’ needs. Data on medications, including ketamine, dexmedetomidine, benzodiazepine, clonidine, and antipsychotics (haloperidol, olanzapine, quetiapine, and risperidone), were obtained from the hospital electronic medical records (EMRs). All notes and medications were time stamped.

### Data definition

Agitation was defined when words suggestive of agitation (e.g., agitation, agitated, combative, restrained, or aggressive) were present in at least one progress note, as previously described.^1 , 2 , 14 , 16^ All notes were logically assessed for words that indicated nonpatient centeredness, resolution, timing and negation (e.g., not, no absent, resolved, etc.).

### Intervention

The use of dexmedetomidine within 30 days of agitation onset was the intervention of interest. During the study period, dexmedetomidine was not used as a sedative agent and was only used for the treatment of agitated *delirium* on the basis of clinical judgment.

The strategy under investigation was based solely on the development of agitation prior to the start of dexmedetomidine therapy and did not include other factors that may have driven the decision to start dexmedetomidine in clinical practice. Different timings of the start of dexmedetomidine following the onset of NLP-identified agitation were also investigated via sensitivity analyses. The comparators were a strategy that never used dexmedetomidine to treat agitation and the natural course, which represents the usual care used for each patient.

### Outcomes

The primary outcome was the resolution of agitation for up to 30 days. Resolution of agitation was defined as the first moment when the patient was free of agitation for at least 12 consecutive hours (no notes with words suggestive of agitation). Discharge from the ICU or death before the resolution of agitation was considered a competing event because it precluded the later occurrence of the primary outcome.

Patients were followed from the onset of agitation (Day 0) until resolution of agitation, ICU discharge, death, or Day 30 in the ICU (in blocks of six hours), whichever occurred first. In all analyses, as in a trial, time zero was the onset of agitation, and the maximum follow-up time was 30 days from agitation onset. The 30-day period was considered because more than 99% of the patients had a length of stay of less than 30 days. The follow-up was divided into blocks of six hours.

The following secondary clinical outcomes were also assessed: 30-day extubation and duration of ventilation (with death before extubation treated as a competing event) and the 30-day tracheostomy rate and time until tracheostomy (only patients receiving MV at agitation onset and with extubation or death without tracheostomy treated as a competing event).

### Analysis plan

In this target trial emulation, dexmedetomidine was a time-dependent exposure because its use was documented at multiple times until Day 30. In addition, there were time-dependent confounders that were affected by prior treatment and that also predicted future treatment with dexmedetomidine and future outcomes, all of which were conditional on past treatment. The use of standard regression methods in this situation results in biased estimates.^17 , 18^ In this setting, target trial emulation using parametric g-formulas is an accepted statistical option. This is because it addresses these points when comparing dynamic treatment regimens ( Supplementary Material ).^19^ In the present study, a g-formula considering a survival outcome was used. In the main analysis, two groups were created. The ‘always received’ group represents a counterfactual scenario where patients are assumed to have received dexmedetomidine from time zero onward, with transition probabilities estimated at each 6-hour time point on the basis of observed covariates. Similarly, the ‘never received’ group represents the scenario in which patients did not receive dexmedetomidine at any time. Competing events were handled using cause-specific hazards.

Target trial emulation creates a framework for likely causal inference from observational data. It explicitly emulates the components of an RCT. It includes screening patients and identifying patients eligible for treatment allocation on the basis of their baseline characteristics and inclusion criteria for the intervention (e.g., agitation). It then follows patients from “randomization emulation time” (time 0 in this case when agitation developed) to the end of planned follow-up and conducts the same analysis as would be conducted for the corresponding target trial.

### Statistical analysis

All continuous data are reported as medians (quartile 25% - quartile 75%), and categorical data are reported as numbers and percentages. The clinical characteristics of the patients were compared among the groups via Fisher’s exact test and the Wilcoxon rank-sum test.

All models were adjusted for the following baseline covariates: age, sex, type of admission (medical or surgical), planned or unplanned admission, the Australian and New Zealand Risk of Death (ANZROD) value after log transformation, days between ICU admission and development of agitation, admission after medical emergency team call, cardiac arrest in the first 24 hours, acute kidney injury at ICU admission, admission diagnosis, ICU source of admission, chronic cardiovascular disease, hepatic failure, metastatic cancer, leukemia, use of renal replacement therapy, use of vasopressors and use of MV. The ANZROD is a validated and accurate predictor of mortality in ICUs in Australia and New Zealand.^20 , 21^ All baseline variables were selected on the basis of clinical relevance, previous evidence and differences between groups. The daily use of ketamine, clonidine, benzodiazepines and antipsychotics and the development of *delirium* ^14^ were included as time-dependent variables. All models included the hour (in blocks of six hours), and the time-dependent intercept was estimated by a smooth function of the day since the beginning of follow-up using natural cubic splines with five knots.

Missing data are reported in table 1S ( Supplementary Material ). Missing baseline data from the first 24 hours after ICU admission were imputed using multiple imputation by chained equations. The list of predictors included the outcome variable, failure time, baseline covariates and laboratory tests. In the case of missing covariate values during the follow-up, the last observed value of a covariate was carried forward, reflecting clinical practice at the bedside.

Subgroup analyses were performed according to age (< 65 *versus* ≥ 65 years), type of admission (medical *versus* surgical), presence of sepsis (yes *versus* no), use of MV (yes *versus* no) and severity of illness (Acute Physiology and Chronic Health Evaluation [APACHE] III < 48 *versus* ≥ 48). A sensitivity analysis assessing the impact of the timing of initiation and duration of treatment with dexmedetomidine on the outcomes was performed ( Supplementary Material ).

As the g-formula estimates marginal risk measures, risk ratios (RR), rather than hazard ratios, are reported because the model focuses on absolute differences in risk over time rather than instantaneous rates of events. All analyses were performed in R Version 4.3.3 (R Foundation for Statistical Computing, Vienna, Austria).

## RESULTS

### Patients

**Figure 1:**
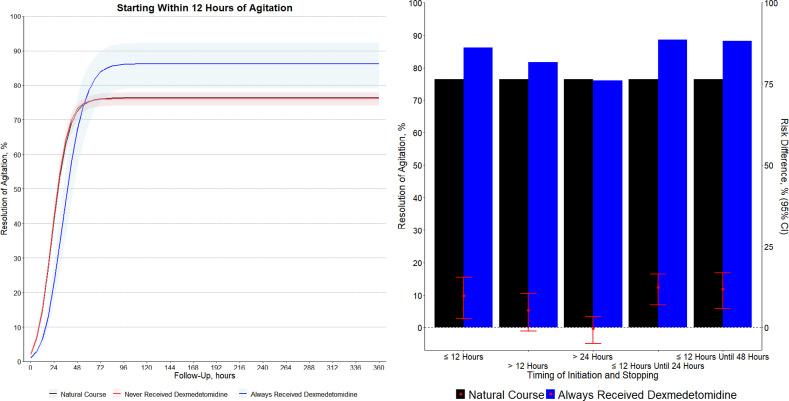
Results of the target trial emulation on the estimated resolution of agitation within 30 days with early use of dexmedetomidine.

From June 2016 until April 2021, 7,525 patients were available for inclusion. Overall, 2,242 patients (29.8%) had at least one episode of agitation in the ICU and were eligible for inclusion. One hundred sixty-seven patients were excluded because they received dexmedetomidine before the episode of agitation, and 23 patients were excluded because of missing hospital outcome data. After such exclusions, 2,052 patients were available for analysis, 314 patients of which were treated with dexmedetomidine ( Figure 1S - Supplementary Material ).

The baseline characteristics of the included patients are shown in table 2 . Patients who received dexmedetomidine were younger, had more severe illness, and were more likely to have unplanned emergency department admissions with a medical diagnosis. During the ICU stay, patients who received dexmedetomidine also received vasopressors, invasive ventilation, and renal replacement therapy more often ( Table 2 ). The median time between ICU admission and the first episode of agitation was 24 (11 - 52) hours, and it was similar between the dexmedetomidine-treated and untreated patients. The median number of notes assessed per patient was 25 (12 - 53), and the median number of notes per patient per day was 9 (7 - 14) (i.e., approximately one note every 2.7 hours).

**Table 2:** Baseline characteristics of study patients

	Overall (n = 2,052)	Dexmedetomidine (n = 314)	No dexmedetomidine (n = 1,738)	p value
Age (years)	61.6 (47.4 - 72.5)	56.9 (43.1 - 67.4)	62.6 (48.3 - 73.5)	< 0.001
Male sex	1,313 (64.0)	224 (71.3)	1,089 (62.7)	0.006
Body mass index (kg/m^2^)	27.5 (23.7 - 31.7)	29.2 (24.1 - 36.3)	27.2 (23.7 - 30.8)	0.039
APACHE III	54.0 (39.0 - 73.0)	62.0 (43.0 - 77.0)	53.5 (39.0 - 72.0)	< 0.001
ANZROD	5.1 (1.2 - 17.8)	6.3 (1.7 - 19.0)	5.0 (1.1 - 17.5)	0.012
Hours between ICU admission and agitation	24.0 (11.0 - 52.2)	22.5 (12.0 - 54.0)	24.0 (11.0 - 52.0)	0.735
Type of admission				0.039
Medical	1,227 (59.8)	204 (65.2)	1,023 (58.9)	
Surgical	824 (40.2)	109 (34.8)	715 (41.1)	
Unplanned admission	1,629 (79.4)	263 (83.8)	1,366 (78.6)	0.041
MET call admission	355 (17.3)	37 (11.8)	318 (18.3)	0.004
Cardiac arrest	79 (3.9)	22 (7.0)	57 (3.3)	0.004
Acute kidney injury	94 (4.6)	25 (8.0)	69 (4.0)	0.005
Admission diagnosis				0.452
Cardiovascular	570 (27.8)	83 (26.5)	487 (28.0)	
Gastrointestinal	379 (18.5)	69 (22.0)	310 (17.8)	
Gynecological	3 (0.1)	0 (0.0)	3 (0.2)	
Hematological	14 (0.7)	4 (1.3)	10 (0.6)	
Metabolic	162 (7.9)	29 (9.3)	133 (7.7)	
Musculoskeletal/skin	30 (1.5)	5 (1.6)	25 (1.4)	
Neurological	261 (12.7)	40 (12.8)	221 (12.7)	
Renal/genitourinary	55 (2.7)	6 (1.9)	49 (2.8)	
Respiratory	304 (14.8)	46 (14.7)	258 (14.8)	
Sepsis	160 (7.8)	17 (5.4)	143 (8.2)	
Trauma	113 (5.5)	14 (4.5)	99 (5.7)	
ICU source of admission				0.002
Emergency department	567 (27.6)	114 (36.3)	453 (26.1)	
Operating room	818 (39.9)	109 (34.7)	709 (40.8)	
Ward	336 (16.4)	38 (12.1)	298 (17.1)	
ICU other hospital	85 (4.1)	19 (6.1)	66 (3.8)	
Other hospital	239 (11.6)	34 (10.8)	205 (11.8)	
ICU same hospital	4 (0.2)	0 (0.0)	4 (0.2)	
Other	3 (0.1)	0 (0.0)	3 (0.2)	
Coexisting disorders				
Diabetes	280 (79.3)	46 (70.8)	234 (81.2)	0.064
Chronic lung disease	194 (9.5)	24 (7.6)	170 (9.8)	0.251
Chronic cardiovascular disease	106 (5.2)	12 (3.8)	94 (5.4)	0.270
Cirrhosis	217 (10.6)	40 (12.7)	177 (10.2)	0.194
Chronic kidney disease	211 (10.3)	32 (10.2)	179 (10.3)	0.999
Chronic immune disease	71 (3.5)	9 (2.9)	62 (3.6)	0.617
Immunosuppression	155 (7.6)	23 (7.3)	132 (7.6)	0.999
Hepatic failure	45 (2.2)	7 (2.2)	38 (2.2)	0.999
Lymphoma	16 (0.8)	3 (1.0)	13 (0.7)	0.724
Metastatic cancer	60 (2.9)	6 (1.9)	54 (3.1)	0.361
Leukemia	38 (1.9)	6 (1.9)	32 (1.8)	0.823
Organ support				
ECMO	11 (0.8)	2 (0.8)	9 (0.7)	0.999
Vasopressor	1,007 (68.6)	217 (88.6)	790 (64.6)	< 0.001
Invasive ventilation	1,483 (80.3)	292 (96.1)	1,191 (77.2)	< 0.001
Noninvasive ventilation	122 (8.3)	22 (9.0)	100 (8.1)	0.613
Renal replacement therapy	272 (17.7)	56 (22.3)	216 (16.8)	0.046
Laboratory tests				
pH	7.4 (7.3 - 7.4)	7.3 (7.3 - 7.4)	7.4 (7.3 - 7.4)	0.002
PaO_2_/FiO_2_	280 (184 - 382)	257 (164 - 360)	284 (190 - 386)	0.004
PaCO_2_ (mmHg)	41 (35 - 46)	42 (36 - 49)	40 (35 - 45)	< 0.001
Lactate (mmol/L)	2.3 (1.6 - 3.7)	2.6 (1.8 - 4.2)	2.3 (1.6 - 3.6)	0.007
Highest creatinine (µmol/L)	96 (71 - 155)	97 (70 - 177)	95 (71 - 154)	0.283
Lowest platelet, x 10 ^9^ /L	175 (116 - 241)	169 (105 - 234)	176 (117 - 242)	0.297
Vital signs				
Lowest MAP (mmHg)	65.0 (58.0 - 71.0)	63.0 (57.0 - 70.0)	65.0 (58.0 - 71.0)	0.099
Highest respiratory rate (breaths/min)	18.0 (15.0 - 24.0)	18.0 (15.0 - 22.0)	18.0 (15.0 - 25.0)	0.095
Highest temperature (°C)	37.3 (36.7 - 37.7)	37.5 (36.8 - 37.9)	37.2 (36.7 - 37.7)	0.033
Urine output (mL)	1,500 (1,061 - 2,150)	1,450 (918 - 2,210)	1,500 (1,085 - 2,140)	0.167

APACHE - Acute Physiology and Chronic Health Evaluation; ANZROD - Australian and New Zealand Risk of Death; ICU - intensive care unit; MET - medical emergency team; ECMO - extracorporeal membrane oxygenation; MAP - mean arterial pressure. Data are the median (interquartile range) or n (%).

### Dexmedetomidine infusion characteristics

**Figure 2:**
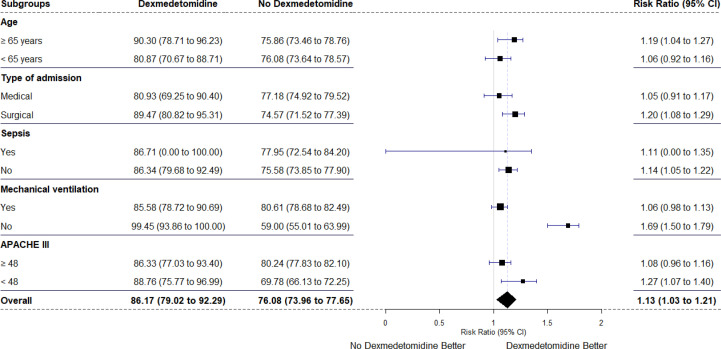
Subgroup analyses of the resolution of agitation within 30 days with early use of dexmedetomidine.

The median number of days between ICU admission and the start of dexmedetomidine treatment was 3 (2 - 6), and the median number of hours between agitation onset and the start of dexmedetomidine treatment was 38 (12 - 92) ( Figure 2S - Supplementary Material ). The mean daily dose of dexmedetomidine was 51 (31 - 78) µg/h (0.56 [0.34 - 0.87] µg/kg/h), the median total dose of dexmedetomidine used per patient was 1966 (597 - 5,035) µg, and dexmedetomidine was used for a median of 2 (1 - 4) days ( Table 3 and Figure 3S - Supplementary Material ). Data on additional medications are also shown in table 3 .

Forest plot showing the impact of dexmedetomidine on the resolution of agitation in different predefined subgroups. The findings were consistent across all the subgroups and particularly strong in the nonventilated group of patients. The sizes of the data markers are proportional to the number of patients included in the analysis.

### Outcomes

Unadjusted outcomes are reported in table 2S ( Supplementary Material ). Resolution of agitation was achieved in 94% of the patients who received dexmedetomidine and 72% of the patients who did not receive dexmedetomidine. At 30 days, 5% of the patients who received dexmedetomidine and 9% of the patients who did not receive dexmedetomidine had died. The time to death was 11 [4 - 14] days in the dexmedetomidine group and 3 [1 - 7] days in the no dexmedetomidine group ( Figure 4S - Supplementary Material ).

### Dexmedetomidine for agitation treatment

Early initiation of dexmedetomidine significantly increased the rate of resolution of agitation within 30 days (RR 1.13 [95%CI 1.03 to 1.21]; risk difference (RD) 9.8% [95% CI, 2.6% to 15.4%]) ( Table 4 , Figure 1 and Table 3S - Supplementary Material ). This possible effect was sustained in situations where the drug was started earlier but was used only for 24 or 48 hours ( Table 3S and Figures 5S and 6S - Supplementary Material ). However, no possible effect of dexmedetomidine was found when the drug was initiated at ≥ 12 hours after the onset of agitation.

**Table 4:** Results of the target trial emulation for different outcomes considering the early use of dexmedetomidine*

	Natural course	Always received dexmedetomidine	Never received dexmedetomidine
Resolution of agitation†			
Crude incidence n (%)	1547 (75.4)	294 (93.6)	1253 (72.1)
Risk %	76.38 (74.17 - 77.92)	86.17 (79.02 - 92.29)	76.08 (73.96 - 77.65)
Risk ratio (95%CI)	1 (Reference)	1.13 (1.03 - 1.21)	1.00 (0.99 - 1.00)
Risk difference (95%CI)	0 (Reference)	9.79 (2.65 - 15.40)	-0.30 (-0.66 - 0.12)
30-day extubation			
Crude incidence n (%)	1032 (96.4)	252 (96.2)	780 (96.5)
Risk %	96.08 (94.54 - 97.20)	99.20 (98.24 - 99.78)	95.72 (94.16 - 96.96)
Risk ratio (95%CI)	1 (Reference)	1.03 (1.02 - 1.04)	1.00 (0.99 - 1.00)
Risk difference (95%CI)	0 (Reference)	3.12 (2.16 - 4.20)	-0.36 (-0.65 - -0.07)
30-day tracheostomy			
Crude incidence n (%)	97 (9.4)	36 (14.1)	61 (7.9)
Risk %	10.49 (8.43 - 12.13)	7.02 (3.32 - 10.48)	11.12 (8.87 - 13.00)
Risk ratio (95%CI)	1 (Reference)	0.67 (0.34 - 0.99)	1.06 (1.00 - 1.12)
Risk difference (95%CI)	0 (Reference)	-3.47 (-7.02 - -0.02)	0.64 (0.00 - 1.26)

95%CI - 95% confidence interval; ICU - intensive care unit.

* Results for the scenario considering the early use of dexmedetomidine (starting within 12 hours of agitation). The results of the other scenarios are reported in the Supplementary Material. The ‘always received’ group represents a counterfactual scenario where patients are assumed to have received dexmedetomidine from time zero onward, with transition probabilities estimated at each 6-hour time point based on observed covariates. Similarly, the ‘never received’ group represents the scenario where patients did not receive dexmedetomidine at any time; † Resolution of agitation was defined as the first moment when the patient was free of agitation for at least 12 consecutive hours. All models adjusted for age, sex, days between intensive care unit admission and development of agitation, type of admission (medical or surgical), planned or unplanned admission, the Australian and New Zealand Risk of Death (ANZROD), admission after medical emergency team call, cardiac arrest in the first 24 hours, acute kidney injury at intensive care unit admission, admission diagnosis, intensive care unit source of admission, chronic cardiovascular disease, hepatic failure, metastatic cancer, leukemia, use of renal replacement therapy, use of vasopressor/inotropes and use of mechanical ventilation. The following time-dependent variables was included: daily use of ketamine, clonidine, benzodiazepines and antipsychotics, and development of *delirium* . All models included the hour in block of six hours, and the time-dependent intercept was estimated by a smooth function of the day since beginning of follow-up using natural cubic splines with five knots.

Left panel, resolution of agitation according to the treatment over time. Estimates from the parametric g-formula. The shaded areas represent the 95% confidence intervals. The numerical results are reported in table 3 . Right panel, resolution of agitation and risk difference between the natural course and the use of dexmedetomidine according to the starting and stopping times. The risk difference is calculated as the difference in risk between a strategy always using dexmedetomidine and the natural course (usual care). The black bars represent the resolution of agitation in the natural course group, and the blue bars represent the resolution of agitation in the dexmedetomidine group. The red circles represent the risk differences between the two treatments, and the red error bars represent the 95% confidence intervals.

Subgroup analyses are reported in figure 2 . Dexmedetomidine increased the resolution of agitation within 30 days in patients older than 65 years, admitted for surgical reasons, with diagnoses other than sepsis, not on MV and with lower APACHE III scores, with a particularly strong signal in nonventilated patients.

### Dexmedetomidine and 30-day extubation and the duration of ventilation

Early initiation of dexmedetomidine increased the chance of extubation by Day 30 and reduced the time until extubation (RR 1.03 [95%CI 1.02 - 1.04]; RD 3.1% [95%CI 2.2% - 4.2%]) ( Table 4 , Table 4S and Figure 7S - Supplementary Material ). In addition, a strategy that simulated a situation where dexmedetomidine was never used demonstrated a reduced chance of being extubated by Day 30 compared with the emulated natural course (RR 1.00 [95%CI 0.99 - 1.00]; RD -0.4% [95%CI -0.6% - -0.1%]) ( Table 4 , Table 4S and Figure 7S - Supplementary Material ). This possible effect was sustained in situations where the drug was started at different timepoints and used for different durations ( Table 4S and Figures 7A and 8S - Supplementary Material ).

### Dexmedetomidine and 30-day tracheostomy

Early initiation of dexmedetomidine reduced the chance of having a tracheostomy by Day 30 (RR 0.67 [95%CI 0.34 - 0.99]; RD -3.5% [95%CI -7.0% to -0.0%]) ( Table 4 , Table 5S and Figures 9S and 10S - Supplementary Material ). In addition, a strategy that simulated a situation where dexmedetomidine was never used increased the chance of having a tracheostomy by Day 30 compared with the natural course (RR 1.06 [95%CI 1.00 - 1.12]; RD 0.6% [95%CI, 0.0% - 1.3%]) ( Table 4 , Table 5S and Figures 9S and 10S - Supplementary Material ).

## DISCUSSION

### Key findings

Using granular EMR data extraction, NLP analytics to identify agitation, and target trial emulation to test for possible causal inferences, we tested whether treatment with dexmedetomidine increased the resolution of agitation. We found that early dexmedetomidine appeared to significantly increase the likelihood of agitation resolution within 30 days of treatment initiation. Moreover, we found that early dexmedetomidine treatment appeared to increase the chance of being extubated, decreased the chance of receiving a tracheostomy and potentially modified the risk of death. Finally, we found that these effects were robust to several sensitivity analyses and were observed in all predefined subgroups.

### Relationship to previous studies

Although dexmedetomidine was tested as a sedative agent in the SPICE-3 clinical trial,^22^ its use for the treatment of agitation in the full ICU population (ventilated and nonventilated patients) has not been explored in RCTs of the general population of patients who might develop agitation in the ICU. However, the Dahlia trial was a double-blind RCT in 15 ICUs in Australia and New Zealand^9^that randomized 74 patients with agitated *delirium* to dexmedetomidine. However, dexmedetomidine was only used in those receiving invasive ventilation who would have been extubated on physiological grounds but could not be extubated because of agitated *delirium* . In such highly selected patients, the use of dexmedetomidine increased the number of ventilator-free days to day seven, decreased the time to extubation, and accelerated the resolution of agitated *delirium* .^9^ Such suggestive observations, however, did not address the effect of dexmedetomidine in more general cohorts of ICU patients, including nonventilated patients.

In addition to the above considerations, the role of dexmedetomidine in critically ill patients remains controversial,^4 , 23^ especially as a sedative agent.^22 , 24^ In this regard, studies thus far have focused on the use of dexmedetomidine either as a sedative agent^25^ or as a means of preventing *delirium* .^26^ Thus, there have been only a few dedicated investigations of the use of dexmedetomidine for the treatment of agitation.^8 , 9 , 27 - 29^ Moreover, few studies have focused on agitation^8 , 9^ or have focused only on postoperative patients.^28^ However, despite these limitations, they have suggested that dexmedetomidine may be associated with a reduced duration of ventilation,^8 , 9 , 28^ a reduced duration of *delirium* ,^8^ and a reduced ICU length of stay.^9^ In addition, studies have suggested that dexmedetomidine may decrease agitation without causing excessive sedation by enabling titration to effect.^30 , 31^ In agreement with such notions, an observational study reported that dexmedetomidine was useful as a rescue treatment for agitation when haloperidol failed.^29^ The approximate 90% success rate with dexmedetomidine in this observational study aligns with our findings of 94% resolution of agitation. Moreover, similar to our study, this signal was observed in nonventilated patients.^29^

### Implications of the study findings

Agitation in ICU patients is associated with an increased risk of self-extubation, ventilator asynchrony, longer ICU stays, and worse overall outcomes, reinforcing the importance of effective management strategies.^32^ While our findings suggest that dexmedetomidine is associated with reduced agitation, it is essential to acknowledge that not all cases require pharmacological intervention. Nonpharmacological strategies, such as optimizing the environment, reorienting patients, and addressing underlying causes such as pain or *delirium* , may be preferable in certain cases, minimizing drug-related side effects and costs.^33^

Our findings suggest that early dexmedetomidine may be an effective agent for controlling agitation in critically ill patients. This success rate exceeds the reported performance of haloperidol. Moreover, this study’s observations also imply that early treatment with dexmedetomidine is desirable. The observation that the effect of dexmedetomidine is more pronounced in older patients, surgical patients without sepsis, and those with lower severity of illness aligns with the findings of previous studies.^22 , 34 - 36^Thus, these findings suggest that these patients, if agitated, may be particularly appropriate targets for early dexmedetomidine, and its alignment with previous studies provides a degree of robustness to our results. Finally, our findings suggest that such control of agitation may be associated with improved patient-centered outcomes and that such effects are consistent across different subgroups and robust to sensitivity analyses.

Importantly, this is not a study of early dexmedetomidine for sedation, as was the case in the SPICE III study.^22^ This was, instead, a study of the treatment of psychomotor agitation. We deliberately targeted agitation and not sedation or the generic diagnosis of *delirium* as the subject of our investigation. We did this for three key reasons: 1) the DAHLIA study demonstrated an effect in patients who could not be extubated because of their psychomotor agitation;^9^ 2) dexmedetomidine, like all other psychotropic medications, has never been shown to be effective in patients with hypoactive *delirium* ; and 3) psychomotor agitation is what puts the patient at risk of self-injury and staff at risk of patient-induced injury. Thus, it is an immediate safety priority.

### Study strengths and limitations

Our study has several strengths. This is the largest assessment of the impact of dexmedetomidine on agitation in critically ill patients. It applies several novel techniques of investigation on the basis of granular electronic data extracted from patient EMRs, the application of NLP, and the conduct of a target trial emulation. It assesses outcomes via g-modeling and adjusts for > 15 baseline and/or time-dependent variables, increasing the likelihood that the observed associations reflect a true effect of the intervention. Finally, it confirms the robustness of its findings in multiple subgroups and sensitivity analyses.

We acknowledge several limitations. Even though target trial emulation is a powerful technique that approximates the conduct of true RCTs in a less biased way than other statistical approaches,^10 - 12^ it cannot yet be used to fully infer causality. Additionally, some potential confounders, such as the pain level, RASS score, and prior history of mental illness or addiction to psychotropic medications, were not included in the models. Thus, our findings remain hypothesis-generating, and the outcomes observed could be explained by other unmeasured effects. Second, this was a single-center study, and the generalizability of our findings is uncertain. However, our resolution of the agitation rate is consistent with that reported in a previous study^29^ and the median dose of dexmedetomidine used is similar to that used in other studies of this drug as a treatment for *delirium* .^9^ We did not systematically collect data on the side effects of dexmedetomidine, such as bradycardia or hypotension. However, the clinical meaning of such pharmacological and physiological effects is unclear, and the clinical outcomes observed support its safety even in a cohort with a high rate of vasopressor therapy. Early dexmedetomidine use was low, which may have limited the precision of the estimates. However, all estimates (for all outcomes and in all sensitivity analyses) point in the same direction, which confirms the consistency of the findings.

## CONCLUSION

In a target trial emulation study using granular electronic medical record data and natural language processing, early treatment with dexmedetomidine was associated with an increased rate of agitation resolution, a higher extubation rate, and a lower tracheostomy rate. These findings were robust to several sensitivity analyses and were observed in all predefined subgroups. These findings support randomized clinical trials of the use of dexmedetomidine for the treatment of within-intensive care unit psychomotor agitation.

**Table 3:** Characteristics of dexmedetomidine and medication use in the included patients

	Dexmedetomidine (n = 314)	No dexmedetomidine (n = 1,738)	p value
Dexmedetomidine			
Hours between agitation and dexmedetomidine	38.0 (12.0 - 91.8)	---	---
Days between ICU admission and dexmedetomidine	3.2 (1.6 - 6.2)	---	---
Duration of dexmedetomidine use (days)	1.7 (0.9 - 3.9)	---	---
Mean daily dose of dexmedetomidine (µg/h)	51.2 (31.2 - 77.7)	---	---
µg/kg/h	0.56 (0.34 - 0.87) (n = 87)	---	---
Total dose of dexmedetomidine (µg)	1,966 (597 - 5,035)	---	---
Other medications			
Any antipsychotic	246 (78.3)	423 (24.3)	< 0.001
Number of doses received	3.0 (1.0 - 7.0)	0.0 (0.0 - 0.0)	< 0.001
Days until first dose	1.5 (0.5 - 3.4)	0.8 (0.4 - 2.4)	< 0.001
Mean dose (mg)	25.0 (16.7 - 41.9)	17.0 (12.5 - 25.0)	< 0.001
Total dose (mg)	208.8 (75.0 - 536.9)	50.5 (25.0 - 175.0)	< 0.001
Atypical antipsychotic*	243 (77.4)	396 (22.8)	< 0.001
Number of doses received	3.0 (1.0 - 7.0)	0.0 (0.0 - 0.0)	< 0.001
Days until first dose	1.6 (0.5 - 3.6)	0.8 (0.4 - 2.7)	0.001
Mean dose (mg)	25.0 (18.8 - 43.6)	20.0 (12.5 - 25.0)	< 0.001
Total dose (mg)	212.5 (75.0 - 546.2)	62.5 (25.0 - 200.0)	< 0.001
Haloperidol	79 (25.2)	108 (6.2)	< 0.001
Number of doses received	0.0 (0.0 - 0.8)	0.0 (0.0 - 0.0)	< 0.001
Days until first dose	4.1 (1.3 - 10.0)	1.8 (0.7 - 4.7)	< 0.001
Mean dose (mg)	2.0 (1.0 - 3.1)	1.5 (1.0 - 2.5)	0.038
Total dose (mg)	4.5 (2.2 - 7.5)	2.5 (1.0 - 5.0)	< 0.001
Benzodiazepines	144 (45.9)	273 (15.7)	< 0.001
Number of doses received	0.0 (0.0 - 2.0)	0.0 (0.0 - 0.0)	< 0.001
Days until first dose	1.9 (0.7 - 5.3)	1.7 (0.4 - 4.7)	0.111
Mean dose (mg)	5.0 (3.0 - 7.3)	5.0 (2.5 - 5.0)	0.014
Total dose (mg)	15.0 (5.0 - 61.9)	5.0 (3.0 - 15.0)	< 0.001
Clonidine	133 (42.4)	225 (12.9)	< 0.001
Number of doses received	3.1 (1.4 - 6.1)	1.3 (0.5 - 3.2)	< 0.001
Days until first dose	0.0 (0.0 - 2.0)	0.0 (0.0 - 0.0)	< 0.001
Mean dose (µg)	50.0 (50.0 - 75.0)	50.0 (50.0 - 75.0)	0.058
Total dose (µg)	250.0 (100.0 - 975.0)	150.0 (50.0 - 350.0)	< 0.001

Data are the median (interquartile range) or n (%). * Includes olanzapine, quetiapine and/or risperidone.

## Supplemental Material

A target trial emulation of dexmedetomidine to treat agitation in the intensive care unit


