# Supplementary material for: A target trial emulation of dexmedetomidine to treat agitation in the intensive care unit
Source: Crit Care Sci. 2025 Mar 14;37:e20250010. doi: 10.62675/2965-2774.20250010 (PMC11991816; doi:10.62675/2965-2774.20250010)
Supplement: Supplementary file 1 [file 2965-2774-ccsci-37-e20250010-suppl.pdf]

Critical Care Science
https://doi.org/10.62675/2965-2774.20250010

SUPPLEMENTARY MATERIAL

A target trial emulation of dexmedetomidine to treat agitation in the intensive care unit

Ary Serpa Neto[1]*, Marcus Young[2]*, Atthaphong Phongphithakchai[1], Akinori Maeda[1], Yukiko Hikasa[1], Nuttapol Pattamin[1], Nuanprae Kitisin[1], Gehan Premaratne[1], Gabriel Chan[1], Joseph Furler[1], Meg Stevens[3], Dinesh Pandey[3], Hossein Jahanabadi[3], Yahya Shehabi[4], Rinaldo Bellomo[5]

METHODS G-FORMULA

Parametric regression models were fitted to estimate the complete joint distribution of the outcome and time-dependent covariates given previous treatment and covariate history. This joint distribution was then used in Monte Carlo simulations to estimate the risk of the outcome if all patients were always treated with dexmedetomidine ('always treated') or if all patients never received dexmedetomidine ('never treated'), and to compare it with the observed risk under natural course (usual care). Non-parametric bootstrapping with 500 resamples was used to estimate 95% confidence intervals (CIs).

G-formula was implemented following three steps: 1) parametric regression models for each of the time-dependent variables as a function of prior risk factor history at each time point; 2) simulation of the course in the ICU for a large pseudo population of patients under the treatment strategy of interest; and 3) bootstrap to obtain 95%CI for the estimated risks of outcomes effect estimates between interventions. All models were adjusted by the baseline covariates and time-dependent covariates described in the main manuscript.

In additional analyses, the impact of timing of initiation and duration of treatment on outcome were assessed. More specifically, the causal effect for the following strategies compared with natural course were assessed: delayed start (12 hours after agitation), late start (24 hours after agitation), early start and short duration (within 12 hours after agitation and for 24 hours use in total), and early start and prolonged duration (within 12 hours after agitation and for 48 hours use in total).

Table 1S - Rate of missing data

	Overall (n = 2,052)	Dexmedetomidine (n = 314)	No dexmedetomidine (n = 1,738)
Age	0 (0.0)	0 (0.0)	0 (0.0)
Male sex	1 (0.0)	0 (0.0)	0 (0.0)
Body mass index	1,808 (88.1)	263 (83.8)	1,545 (88.9)
APACHE III	1 (0.0)	1 (0.3)	0 (0.0)
ANZROD	6 (0.3)	1 (0.3)	5 (0.3)
Type of admission	1 (0.0)	1 (0.3)	0 (0.0)
Planned admission	0 (0.0)	0 (0.0)	0 (0.0)
MET call admission	0 (0.0)	0 (0.0)	0 (0.0)
Cardiac arrest	1 (0.0)	0 (0.0)	1 (0.1)
Acute renal failure	14 (0.7)	1 (0.3)	13 (0.7)
Admission diagnosis	1 (0.0)	1 (0.3)	0 (0.0)
ICU source of admission	0 (0.0)	0 (0.0)	0 (0.0)

Continue...

This is an open access article under the CC BY license (https://creativecommons.org/licenses/by/4.0/).

...continuation

Coexisting disorders			
Diabetes	1,699 (82.8)	249 (79.3)	1,540 (83,4)
Chronic lung disease	0 (0.0)	0 (0.0)	0 (0.0)
Chronic cardiovascular disease	0 (0.0)	0 (0.0)	0 (0.0)
Cirrhosis	0 (0.0)	0 (0.0)	0 (0.0)
Chronic kidney disease	0 (0.0)	0 (0.0)	0 (0.0)
Immunosuppression	0 (0.0)	0 (0.0)	0 (0.0)
Hepatic failure	0 (0.0)	0 (0.0)	0 (0.0)
Lymphoma	0 (0.0)	0 (0.0)	0 (0.0)
Metastatic cancer	0 (0.0)	0 (0.0)	0 (0.0)
Leukemia	0 (0.0)	0 (0.0)	0 (0.0)
Organ support			
ECMO	586 (28.6)	70 (22.3)	516 (29.7)
Vasopressor or inotropes	585 (28.5)	69 (22.0)	516 (29.7)
Invasive ventilation	205 (10.0)	10 (3.2)	195 (11.2)
Noninvasive ventilation	578 (28.2)	70 (22.3)	508 (29.2)
Renal replacement therapy	518 (25.2)	63 (20.1)	455 (26.2)
Laboratory tests			
pH	187 (9.1)	13 (4.1)	174 (10.0)
PaO_2/FiO_2	187 (9.1)	13 (4.1)	174 (10.0)
$PaCO_2$	191 (9.3)	14 (4.5)	177 (10.2)
Lactate	664 (31.9)	81 (25.8)	573 (33.0)
Highest creatinine	302 (14.7)	45 (14.3)	257 (14.8)
Lowest platelet	302 (14.7)	46 (14.6)	256 (14.7)
Vital signs			
Lowest MAP	29 (1.4)	6 (1.9)	23 (1.3)
Highest respiratory rate	19 (0.9)	4 (1.3)	15 (0.9)
Highest temperature	16 (0.8)	4 (1.3)	12 (0.7)
Urine output	526 (25.6)	65 (20.7)	461 (26.5)
Use of medications			
Ketamine	0 (0.0)	0 (0.0)	0 (0.0)
Antipsychotic	0 (0.0)	0 (0.0)	0 (0.0)
Benzodiazepines	0 (0.0)	0 (0.0)	0 (0.0)
Clonidine	0 (0.0)	0 (0.0)	0 (0.0)
Primary outcome			
Resolution of agitation	0 (0.0)	0 (0.0)	0 (0.0)
Secondary outcomes 30-day extubation			
Duration of ventilation	0 (0.0)	0 (0.0)	0 (0.0)
Tracheostomy	0 (0.0)	0 (0.0)	0 (0.0)
Days until tracheostomy	0 (0.0)	0 (0.0)	0 (0.0)
Additional clinical outcomes			
30-day mortality	0 (0.0)	0 (0.0)	0 (0.0)
ICU length of stay	0 (0.0)	0 (0.0)	0 (0.0)

Table 2S - Clinical outcomes in included patients

	Overall (n = 2,052)	Dexmedetomidine (n = 314)	No dexmedetomidine (n = 1,738)
Primary outcome			
Resolution of agitation	1,547 (75.4)	294 (93.6)	1,253 (72.1)
Days until resolution	1.0 (0.9 - 1.3)	1.2 (1.0 -1.9)	1.0 (0.9 - 1.2)
Secondary outcomes			
30-day mortality	167 (8.1)	16 (5.1)	151 (8.7)
Days until mortality	3.3 (1.1 - 7.7)	10.9 (4.8 - 13.9)	3.1 (1.0 - 7.2)
30-day extubation	1,032 (96.4)	252 (96.2)	780 (96.5)
Duration of ventilation (days)	1.9 (0.6 - 5.0)	3.4 (1.5 - 7.8)	1.5 (0.5 - 4.0)
Additional clinical outcomes			
30-day tracheostomy	97 (9.4)	36 (14.1)	61 (7.9)
Days until tracheostomy	8.5 (5.2 - 13.5)	11.2 (7.3 - 13.8)	7.8 (4.6 - 12.8)
ICU length of stay (days)	2.5 (1.0 - 6.2)	6.9 (3.8 - 13.7)	2.1 (0.8 - 4.7)

ICU - intensive care unit; NLP-Dx-BD is natural language process diagnosed behavior disturbance. * Duration of ventilation reported only in patients who received ventilation. Data are n (%) or median (interquartile range).

Table 3S - Results of the target trial emulation of dexmedetomidine for the treatment of agitation*

	Resolution of agitation % (95%CI)	Risk ratio (95%CI)	Risk difference (95%CI)
Starting within 12 hours of agitation			
Natural course	76.38 (74.17 to 77.92)	1 (Reference)	0 (Reference)
Never received dexmedetomidine	76.08 (73.96 to 77.65)	1.00 (0.99 to 1.00)	-0.30 (-0.66 to 0.12)
Always received dexmedetomidine	86.17 (79.02 to 92.29)	1.13 (1.03 to 1.21)	9.79 (2.65 to 15.40)
Starting 12 hours after agitation			
Natural course	76.38 (74.17 to 77.92)	1 (Reference)	0 (Reference)
Never received dexmedetomidine	76.07 (73.83 to 77.60)	1.00 (0.99 to 1.00)	-0.31 (-0.58 to 0.02)
Always received dexmedetomidine	81.70 (75.46 to 87.06)	1.07 (0.99 to 1.14)	5.32 (-1.10 to 10.39)
Starting 24 hours after agitation			
Natural course	76.38 (74.17 to 77.92)	1 (Reference)	0 (Reference)
Never received dexmedetomidine	76.21 (74.01 to 77.74)	1.00 (0.99 to 1.00)	-0.17 (-0.40 to 0.12)
Always received dexmedetomidine	76.04 (71.05 to 80.03)	1.00 (0.94 to 1.05)	-0.34 (-4.99 to 3.45)
Starting within 12 hours of agitation and stopping at 24 hours			
Natural course	76.38 (74.17 to 77.92)	1 (Reference)	0 (Reference)
Never received dexmedetomidine	75.98 (73.82 to 77.54)	0.99 (0.99 to 1.00)	-0.40 (-0.69 to -0.06)
Always received dexmedetomidine	88.64 (82.98 to 92.70)	1.16 (1.09 to 1.22)	12.26 (6.97 to 16.42)
Starting within 12 hours of agitation and stopping at 48 hours			
Natural course	76.38 (74.17 to 77.92)	1 (Reference)	0 (Reference)
Never received dexmedetomidine	76.05 (73.92 to 77.61)	1.00 (0.99 to 1.00)	-0.33 (-0.67 to 0.08)
Always received dexmedetomidine	88.24 (82.36 to 93.41)	1.16 (1.08 to 1.22)	11.86 (5.85 to 16.78)

95%CI - 95% confidence interval. * Resolution of agitation was defined as the first moment when the patient was free of agitation for at least 12 consecutive hours. All models adjusted for age, sex, days between intensive care unit admission and development of agitation, type of admission (medical or surgical), planned or unplanned admission, the Australian and New Zealand Risk of Death (ANZROD), admission after medical emergency team call, cardiac arrest in the first 24 hours, acute kidney injury at intensive care unit admission, admission diagnosis, intensive care unit source of admission, chronic cardiovascular disease, hepatic failure, metastatic cancer, leukemia, use of renal replacement therapy, use of vasopressor/inotropes and use of mechanical ventilation. The following time-dependent variables were included: daily use of ketamine, clonidine, benzodiazepines and antipsychotics, and development of delirium. All models included the hour in block of six hours, and the time-dependent intercept was estimated by a smooth function of the day since beginning of follow-up using natural cubic splines with five knots.

Table 4S - Results of the target trial emulation of dexmedetomidine for 30-day extubation

	30-day extubation, % (95% CI)	Risk ratio (95%CI)	Risk difference (95%CI)
Starting within 12 hours of agitation			
Natural course	96.08 (94.54 to 97.20)	1 (Reference)	0 (Reference)
Never received dexmedetomidine	95.72 (94.16 to 96.96)	1.00 (0.99 to 1.00)	-0.36 (-0.65 to -0.07)
Always received dexmedetomidine	99.20 (98.24 to 99.78)	1.03 (1.02 to 1.04)	3.12 (2.16 to 4.20)
Starting 12 hours after agitation			
Natural course	96.08 (94.54 to 97.20)	1 (Reference)	0 (Reference)
Never received dexmedetomidine	95.71 (94.16 to 96.97)	1.00 (0.99 to 1.00)	-0.37 (-0.64 to -0.13)
Always received dexmedetomidine	99.05 (98.10 to 99.62)	1.03 (1.02 to 1.04)	2.97 (2.04 to 3.97)
Starting 24 hours after agitation			
Natural course	96.08 (94.54 to 97.20)	1 (Reference)	0 (Reference)
Never received dexmedetomidine	95.72 (94.17 to 96.97)	1.00 (0.99 to 1.00)	-0.35 (-0.62 to -0.15)
Always received dexmedetomidine	98.91 (97.91 to 99.53)	1.03 (1.02 to 1.04)	2.83 (1.92 to 3.79)
Starting within 12 hours of agitation and stopping at 24 hours			
Natural course	96.08 (94.54 to 97.20)	1 (Reference)	0 (Reference)
Never received dexmedetomidine	95.95 (94.44 to 97.12)	1.00 (1.00 to 1.00)	-0.13 (-0.23 to -0.05)
Always received dexmedetomidine	97.22 (95.92 to 98.22)	1.01 (1.01 to 1.02)	1.14 (0.54 to 1.83)
Starting within 12 hours of agitation and stopping at 48 hours			
Natural course	96.08 (94.54 to 97.20)	1 (Reference)	0 (Reference)
Never received dexmedetomidine	95.91 (94.39 to 97.09)	1.00 (1.00 to 1.00)	-0.17 (-0.29 to -0.06)
Always received dexmedetomidine	97.40 (96.14 to 98.35)	1.01 (1.01 to 1.02)	1.32 (0.66 to 2.00)

95% CI - 95% confidence interval. All models adjusted for age, sex, days between intensive care unit admission and development of agitation, type of admission (medical or surgical), planned or unplanned admission, the Australian and New Zealand Risk of Death (ANZROD), admission after medical emergency team call, cardiac arrest in the first 24 hours, acute kidney injury at intensive care unit admission, admission diagnosis, intensive care unit source of admission, chronic cardiovascular disease, hepatic failure, metastatic cancer, leukemia, use of renal replacement therapy, use of vasopressor/inotropes and use of mechanical ventilation. The following time-dependent variables was included: daily use of ketamine, clonidine, benzodiazepines and antipsychotics, and development of delirium. All models included the hour in block of six hours, and the time-dependent intercept was estimated by a smooth function of the day since beginning of follow-up using natural cubic splines with five knots.

Table 5S - Results of the target trial emulation of dexmedetomidine for 30-day tracheostomy rate

	30-day tracheostomy, % (95%CI)	Risk ratio (95%CI)	Risk difference (95%CI)
Starting within 12 hours of agitation			
Natural course	10.49 (8.43 to 12.13)	1 (Reference)	0 (Reference)
Never received dexmedetomidine	11.12 (8.87 to 13.00)	1.06 (1.00 to 1.12)	0.64 (0.00 to 1.26)
Always received dexmedetomidine	7.02 (3.32 to 10.48)	0.67 (0.34 to 0.99)	-3.47 (-7.02 to -0.02)
Starting 12 hours after agitation			
Natural course	10.49 (8.43 to 12.13)	1 (Reference)	0 (Reference)
Never received dexmedetomidine	11.11 (8.86 to 12.97)	1.06 (1.00 to 1.12)	0.62 (-0.01 to 1.24)
Always received dexmedetomidine	7.34 (3.56 to 10.68)	0.70 (0.37 to 1.01)	-3.14 (-6.63 to 0.08)
Starting 24 hours after agitation			
Natural course	10.49 (8.43 to 12.13)	1 (Reference)	0 (Reference)
Never received dexmedetomidine	11.06 (8.83 to 12.92)	1.06 (1.00 to 1.11)	0.58 (-0.03 - 1.16)
Always received dexmedetomidine	7.68 (3.83 to 10.96)	0.73 (0.40 to 1.03)	-2.81 (-6.26 to 0.25)
Starting within 12 hours of agitation and stopping at 24 hours			
Natural course	10.49 (8.43 to 12.13)	1 (Reference)	0 (Reference)
Never received dexmedetomidine	10.70 (8.58 to 12.42)	1.02 (1.01 to 1.04)	0.22 (0.05 to 0.39)
Always received dexmedetomidine	8.20 (6.21 to 10.32)	0.78 (0.65 to 0.94)	-2.29 (-3.75 to -0.52)
Starting within 12 hours of agitation and stopping at 48 hours			
Natural course	10.49 (8.43 to 12.13)	1 (Reference)	0 (Reference)
Never received dexmedetomidine	10.80 (8.66 to 12.56)	1.03 (1.01 to 1.05)	0.31 (0.06 to 0.55)
Always received dexmedetomidine	7.95 (5.93 to 10.18)	0.76 (0.61 to 0.94)	-2.54 (-4.10 to -0.58)

95% CI - 95% confidence interval. All models adjusted for age, sex, days between intensive care unit admission and development of agitation, type of admission (medical or surgical), planned or unplanned admission, the Australian and New Zealand Risk of Death (ANZROD), admission after medical emergency team call, cardiac arrest in the first 24 hours, acute kidney injury at intensive care unit admission, admission diagnosis, intensive care unit source of admission, chronic cardiovascular disease, hepatic failure, metastatic cancer, leukemia, use of renal replacement therapy, use of vasopressor/inotropes and use of mechanical ventilation. The following time-dependent variables was included: daily use of ketamine, clonidine, benzodiazepines and antipsychotics, and development of delirium. All models included the hour in block of six hours, and the time-dependent intercept was estimated by a smooth function of the day since beginning of follow-up using natural cubic splines with five knots.

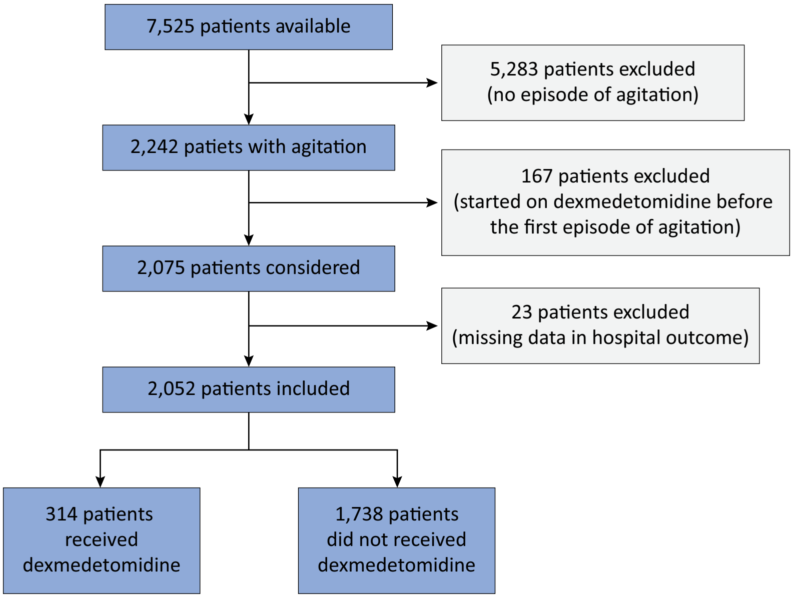

Figure 1S - Flowchart of patient inclusion.

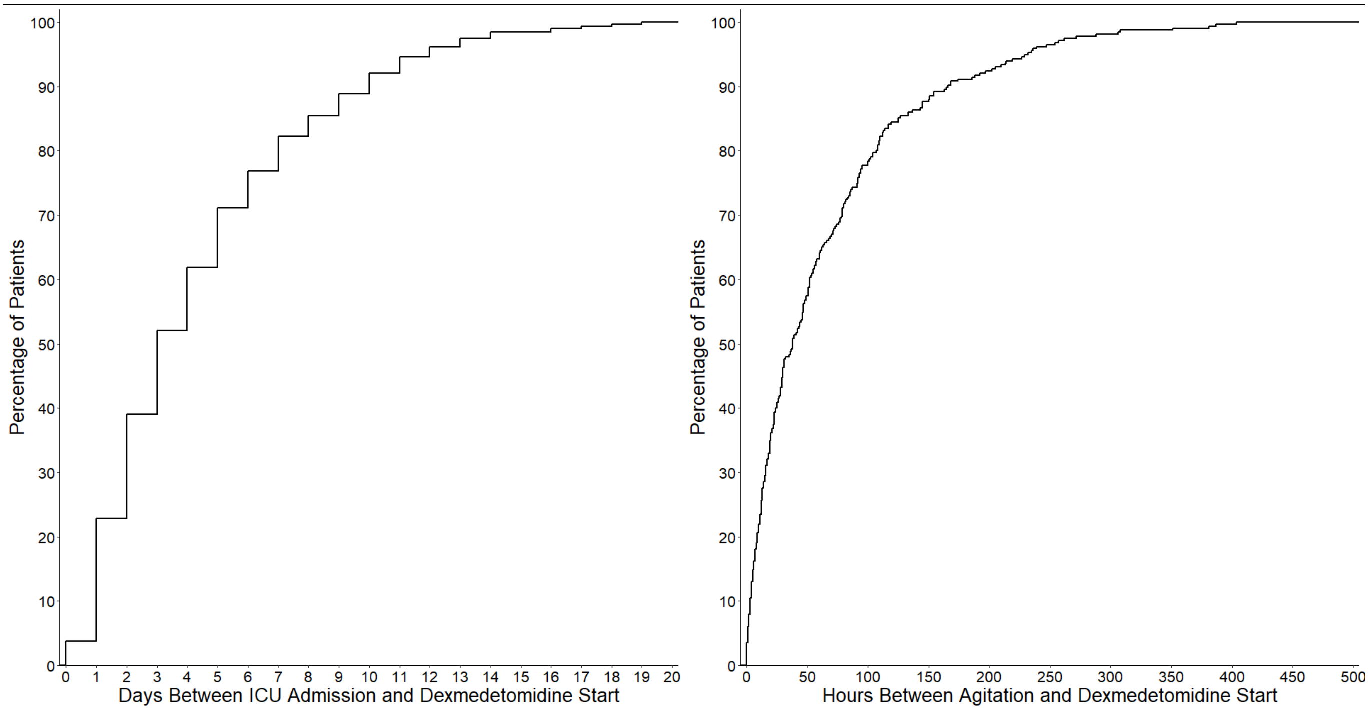

Figure 2S - Days until dexmedetomidine start.
ICU - intensive care unit.

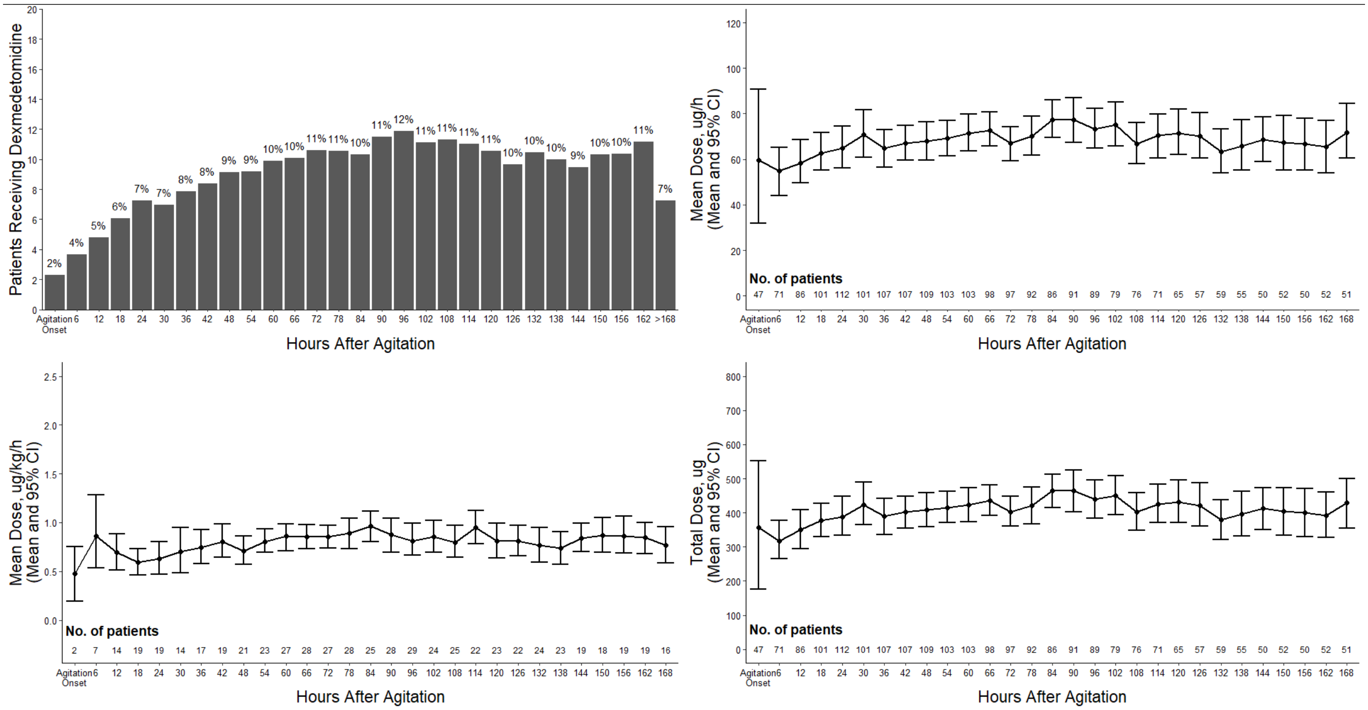

Figure 3S - Use of dexmedetomidine during intensive care unit stay.

Top left, percentage of patients receiving dexmedetomidine during follow-up after agitation. Top right, mean daily dose of dexmedetomidine in μg/h. Circles are mean and error bars are 95% confidence intervals. Bottom left, mean daily dose of dexmedetomidine per kilogram in μg/kg/h. Circles are mean and error bars are 95% confidence intervals. Less patients are reported due to missing data in weight. Bottom right, total daily dose of dexmedetomidine in μg. Circles are mean and error bars are 95% confidence interval.

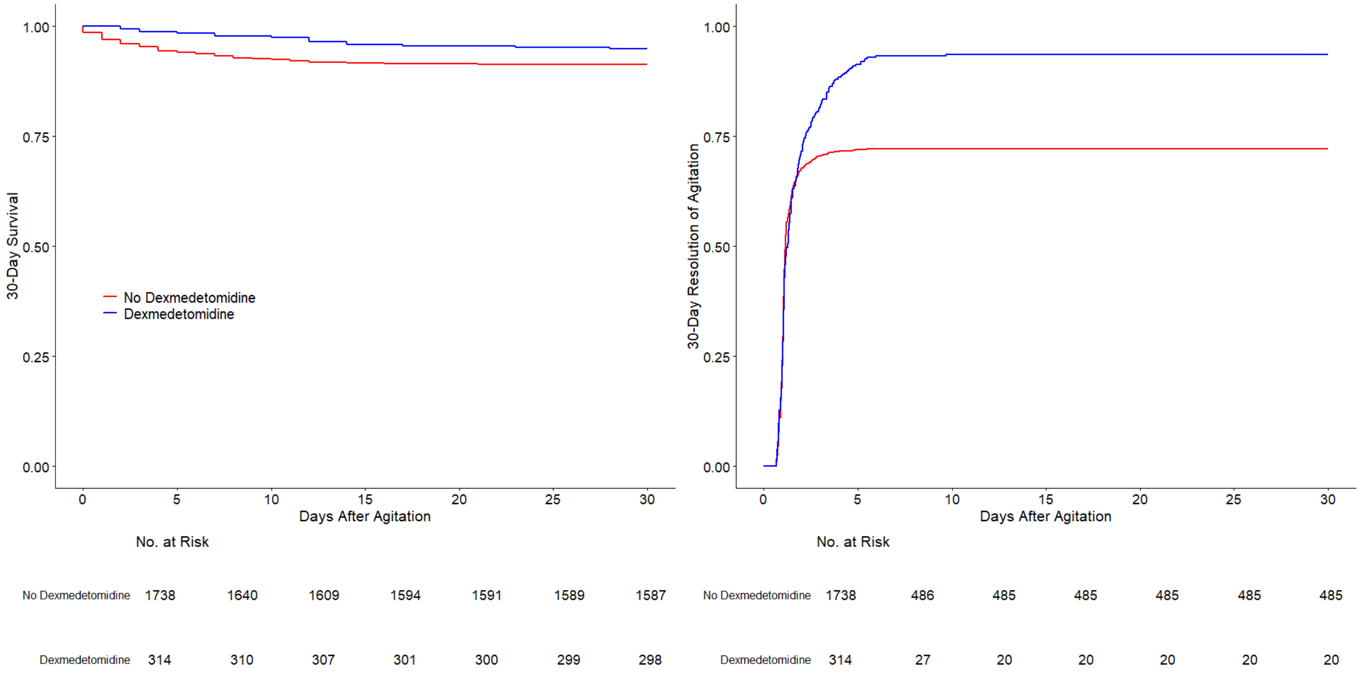

Figure 4S - 30-day survival and resolution of agitation according to the use of dexmedetomidine.

Kaplan-Meier curves with patients who did not develop the outcome of interest censored at day 30.

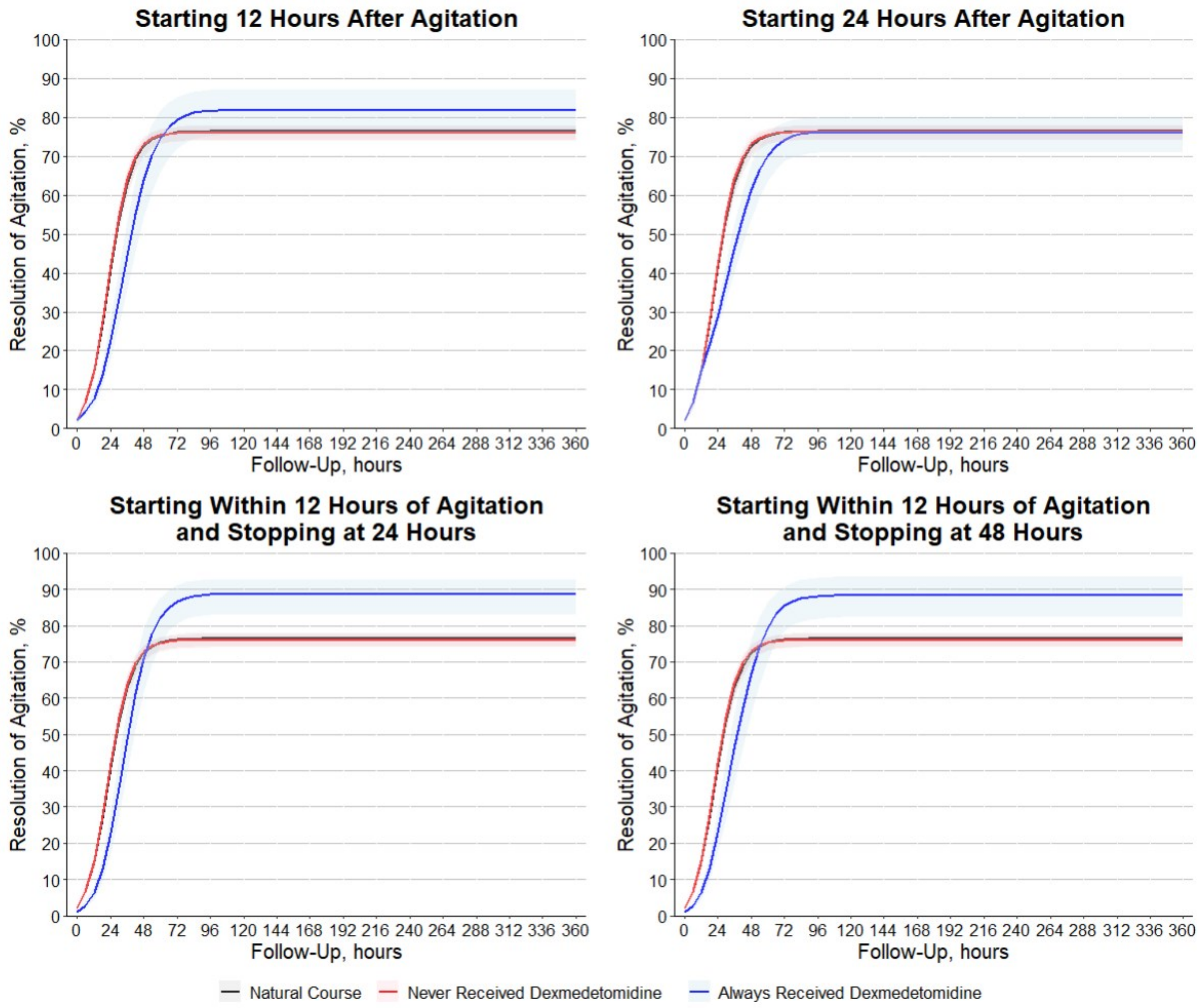

Figure 5S - Results of target trial emulation on estimated resolution of agitation within 30 days under different treatment strategies with varying initiation timing and duration of treatment.

Estimates from parametric g-formula. Shaded areas are 95% confidence interval. Numerical results are reported in table 3S.

UP

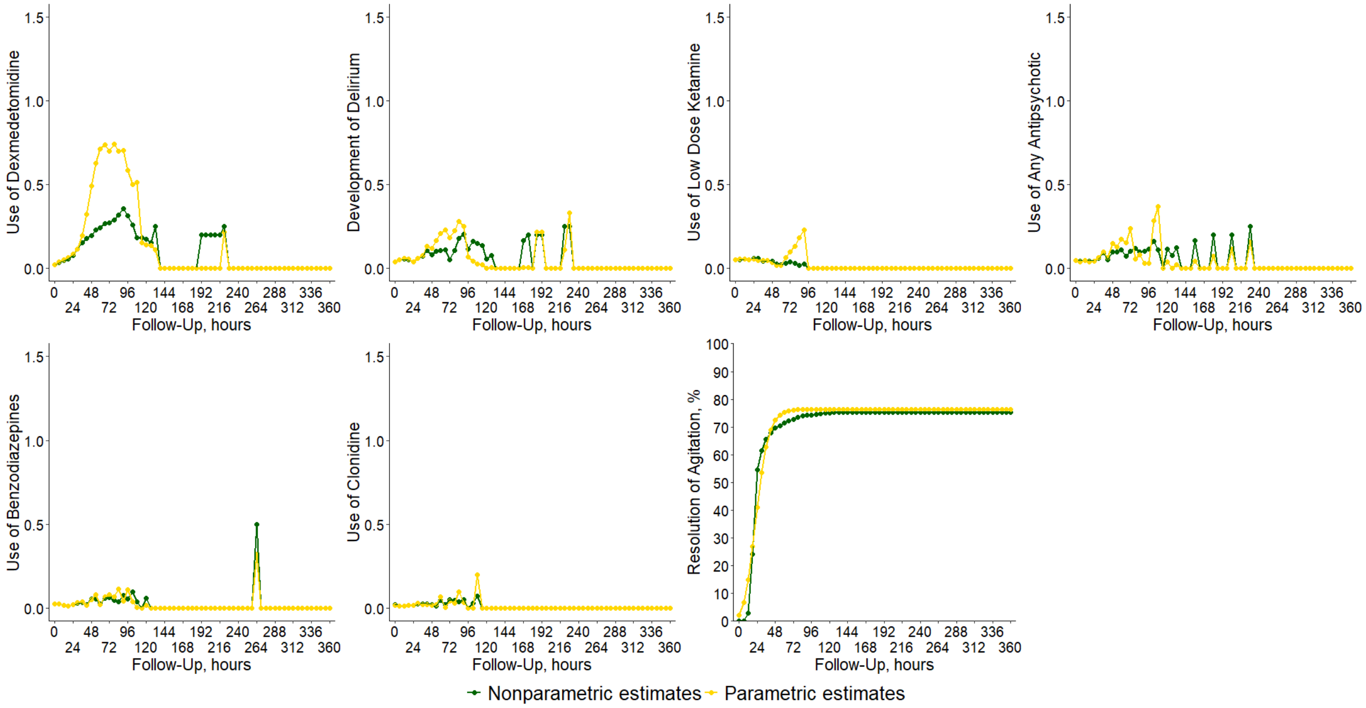

Figure 6S - Parametric versus nonparametric estimate of covariates and resolution of agitation under natural course during follow-up.
Comparable parametric versus nonparametric estimates of the covariates were reported for the natural course, suggesting correct model specification for the parametric g-formula.

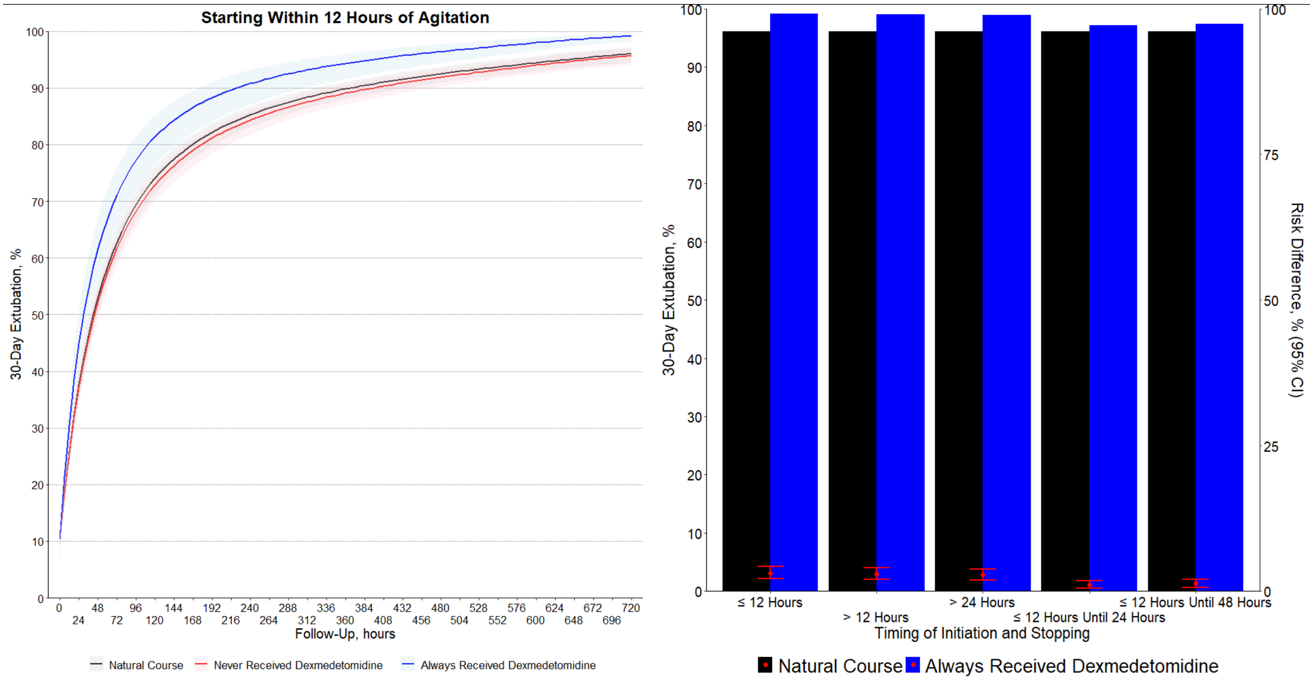

Figure 7S - Results of target trial emulation on estimated 30-day extubation with early use of dexmedetomidine.
Left panel, 30-day extubation according to treatment over time. Estimates from parametric g-formula. Shaded areas are 95% confidence interval. Numerical results are reported in eTable 4. Right panel, 30-day extubation and risk difference between natural course and use of dexmedetomidine according to starting and stopping time. Risk difference calculated as the difference in risk between a strategy always using dexmedetomidine and the natural course (usual care). Black bars are the resolution of agitation in natural course and blue bars in dexmedetomidine group. Red circles are the risk difference and red error bars are the 95% confidence interval.

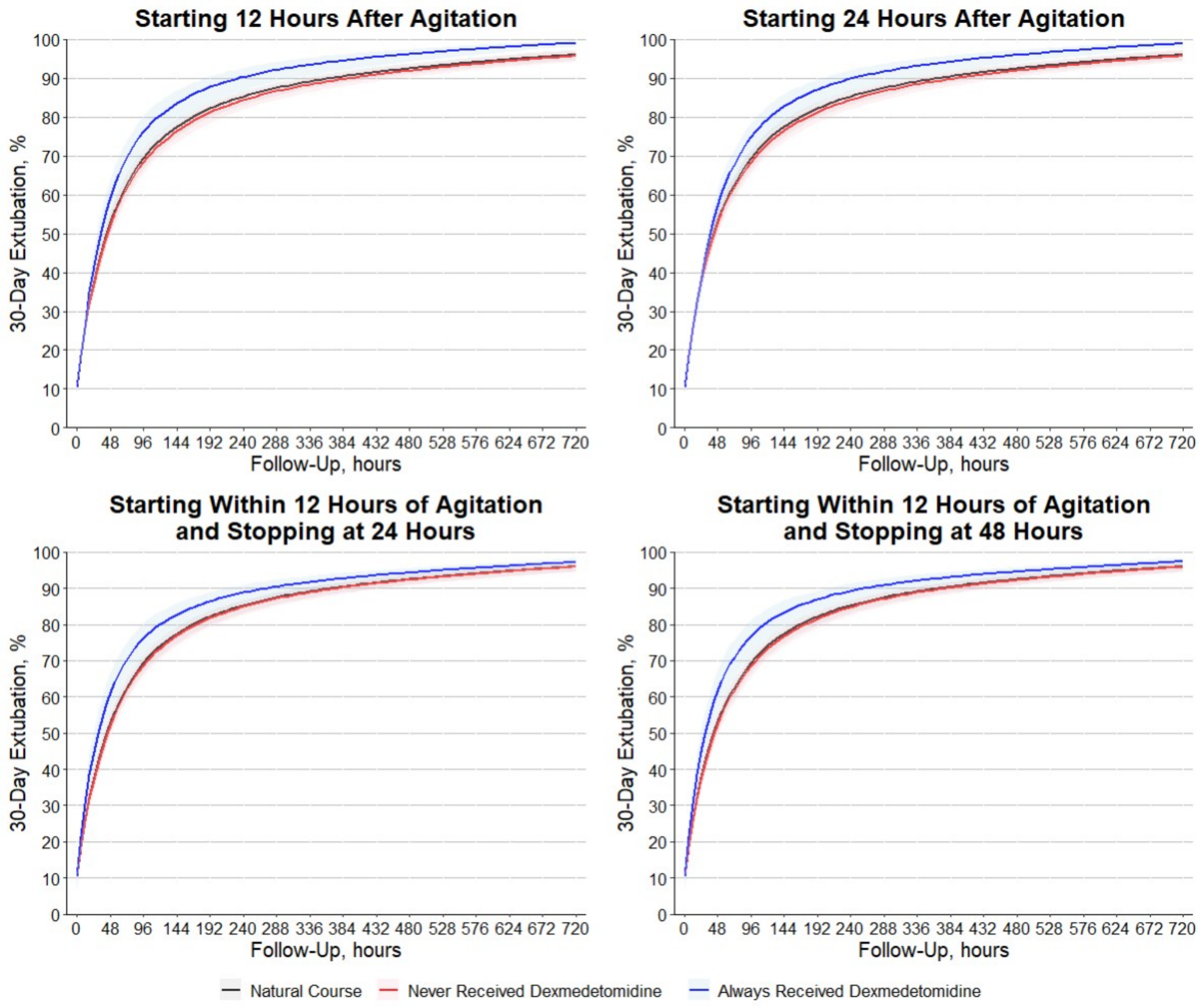

Figure 8S - Results of target trial emulation on estimated 30-day extubation under different treatment strategies with varying initiation timing and duration of treatment.

Estimates from parametric g-formula. Shaded areas are 95% confidence interval. Numerical results are reported in table 4S.

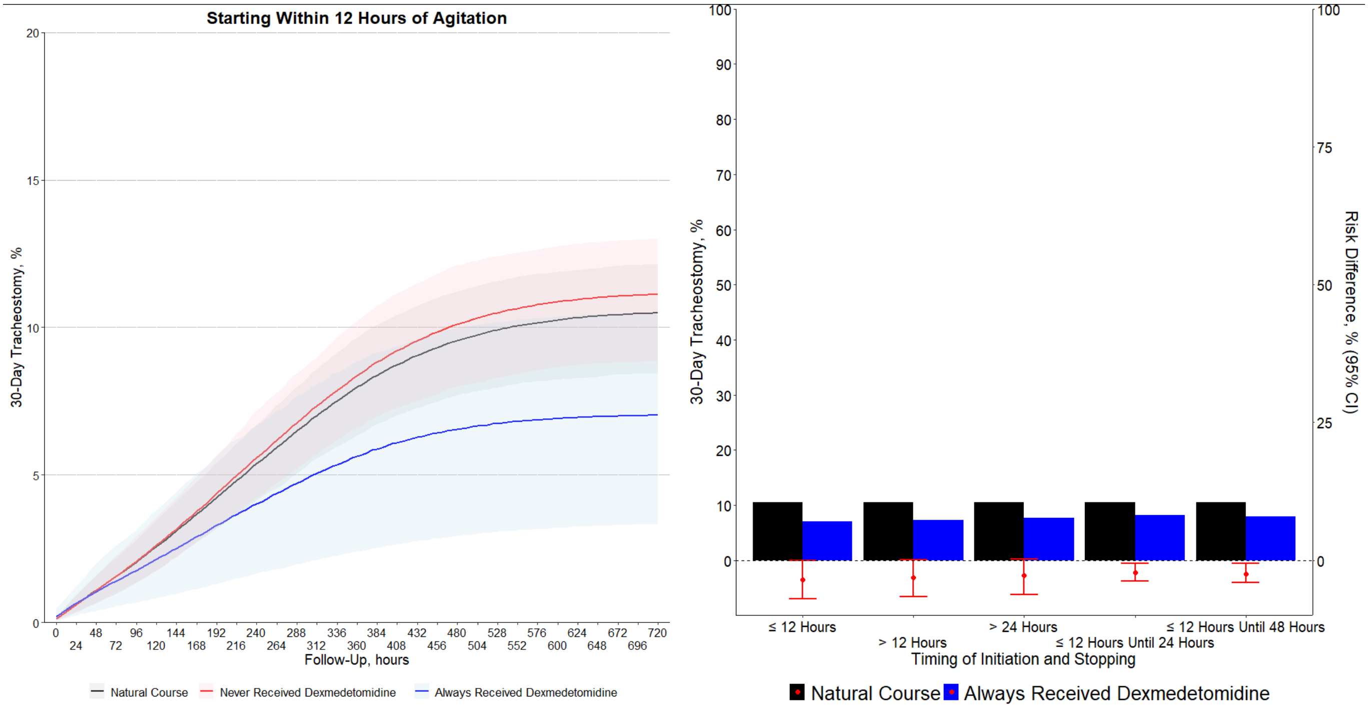

Figure 9S - Results of target trial emulation on estimated 30-day tracheostomy with early use of dexmedetomidine.

Left panel, 30-day tracheostomy according to treatment over time. Estimates from parametric g-formula. Shaded areas are 95% confidence interval. Numerical results are reported in eTable 5. Right panel, 30-day tracheostomy and risk difference between natural course and use of dexmedetomidine according to starting and stopping time. Risk difference calculated as the difference in risk between a strategy always using dexmedetomidine and the natural course (usual care). Black bars are the resolution of agitation in natural course and blue bars in dexmedetomidine group. Red circles are the risk difference and red error bars are the 95% confidence interval.

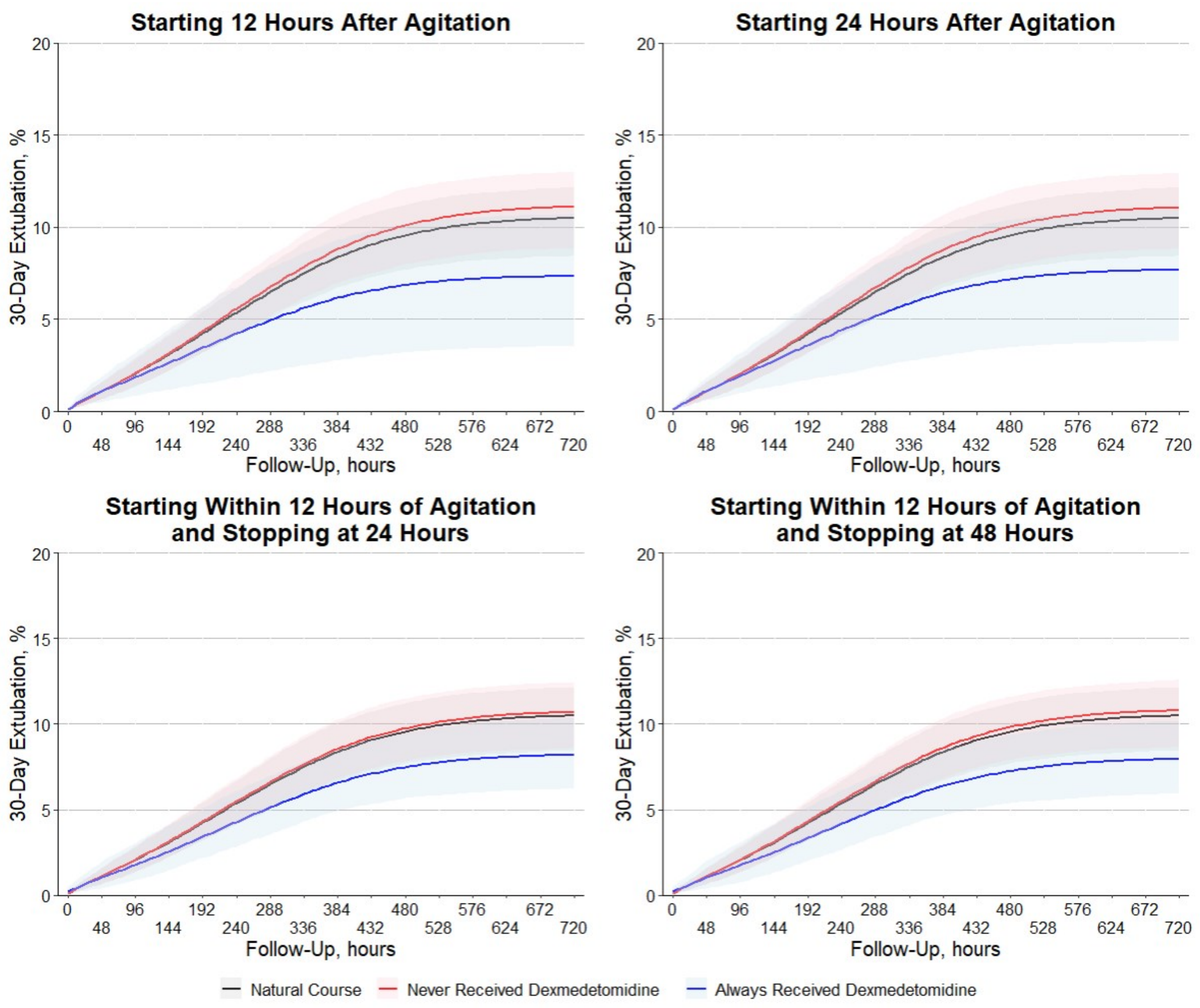

Figure 10S - Results of target trial emulation on estimated 30-day tracheostomy under different treatment strategies with varying initiation timing and duration of treatment.

Estimates from parametric g-formula. Shaded areas are 95% confidence interval. Numerical results are reported in table 5S.
